# Modeling and Characterization of Traffic Flows in Urban Environments

**DOI:** 10.3390/s18072020

**Published:** 2018-06-23

**Authors:** Jorge Luis Zambrano-Martinez, Carlos T. Calafate, David Soler, Juan-Carlos Cano, Pietro Manzoni

**Affiliations:** 1 Department of Computer Engineering (DISCA), Universitat Politècnica de València, 46022 Valencia, Spain; calafate@disca.upv.es (C.T.C.); jucano@disca.upv.es (J.-C.C.); pmanzoni@disca.upv.es (P.M.); 2 Institute of Multidisciplinary Mathematics (IMM), Universitat Politècnica de València, 46022 Valencia, Spain; dsoler@mat.upv.es

**Keywords:** autonomous vehicles, intelligent transportation systems, SUMO, DFROUTER, traffic prediction, traffic behavior, logistic regression, clustering, urban traffic, Valencia

## Abstract

Currently, one of the main challenges faced in large metropolitan areas is traffic congestion. To address this problem, adequate traffic control could produce many benefits, including reduced pollutant emissions and reduced travel times. If it were possible to characterize the state of traffic by predicting future traffic conditions for optimizing the route of automated vehicles, and if these measures could be taken to preventively mitigate the effects of congestion with its related problems, the overall traffic flow could be improved. This paper performs an experimental study of the traffic distribution in the city of Valencia, Spain, characterizing the different streets of the city in terms of vehicle load with respect to the travel time during rush hour traffic conditions. Experimental results based on realistic vehicular traffic traces from the city of Valencia show that only some street segments fall under the general theory of vehicular flow, offering a good fit using quadratic regression, while a great number of street segments fall under other categories. Although in some cases such discrepancies are related to lack of traffic, injecting additional vehicles shows that significant mismatches still persist. Thus, in this paper we propose an equation to characterize travel times over a segment belonging to the sigmoid family; specifically, we apply logistic regression, being able to significantly improve the curve fitting results for most of the street segments under analysis. Based on our regression results, we performed a clustering analysis of the different street segments, showing that they can be classified into three well-defined categories, which evidences a predictable traffic distribution using the logistic regression throughout the city during rush hours, and allows optimizing the traffic for automated vehicles.

## 1. Introduction

One of the critical problems for city authorities is the increase in carbon dioxide (CO${}_{2}$) emissions caused by traffic congestion, resulting in increased travel times of vehicles being associated with delays and inefficient use of fuel [1]. As we gradually move towards a new paradigm centered on automated vehicles, we are able to empower traffic administrators with more sophisticated ways of regulating traffic. The usual strategies to address this problem are based on semaphore timing regulations. For instance, PDDL+ [2] is a planning approach for urban traffic control that efficiently reduces congestion of specific roads. Another solution is the AI system proposed by Pozanco et al. [3] that relies on a declarative automated planning strategy to generate control plans when the default behavior should be overridden. Another strategy is the deployment of traffic agents on site [4] that are independently controlled by an on-line scheduling agent that is able to optimize the movement and control of the traffic on intersections using schedule generated. Among these novel techniques of handling traffic, centralized route management emerges as a solution offering authorities full control of the traffic flow [5], thus allowing traffic optimization to reach maximum levels of effectiveness by deciding the route to be followed by each individual vehicle. By gaining a priori knowledge of the traffic congestion status, it becomes possible to optimize the route of new vehicles, especially in the case of automated vehicles, which can rely on a centralized trusted agent to indicate the most adequate route for each vehicle. An example of such approach was proposed by Chrpa et al. [6], a solution that is able to perform intelligent routing of road traffic through congested urban areas to alleviate congestion, and thereby improve the overall traffic flow. When progressing towards these new traffic management paradigms, it becomes mandatory to gain full awareness of the behavior of the different street segments of a city in terms of how travel time can vary depending on the number of vehicles simultaneously traveling on a particular segment. In this work, we rely on realistic traffic models describing the traffic behavior in the city of Valencia during rush hours, as detailed in our previous work [7]. In particular, starting from induction loops measurements made available by the City Hall of Valencia [8], and by using the DFROUTER [9] tool, along with a heuristic that iteratively refines the output produced by this tool, we generate an Origin–Destination (O-D) traffic matrix that resembles the real traffic distribution. This way we demonstrate the degrees of expected congestion, and also the impact of unpredictable events that cause additional traffic in the city [10]. In this work, we extend our previous contribution by analyzing, modeling and characterizing how traffic becomes distributed along a city, gathering details about the number of vehicles traveling along the different street segments, as well as their travel times [11], using the reference traffic as input load to the Simulation of Urban MObility (SUMO) tool [12]. Post-processing of the gathered data allows merging segments when excessive fragmentation is detected, characterizing the different streets in terms of travel time behavior under variable traffic loads. In particular, we sample the travel times of vehicles entering a segment, along with the number of vehicles already in that segment, allowing to extract a relation between street occupation and delay. Through regression analysis, we show that a quadratic adjustment, despite agreeing with traffic flow theory [13], is not adequate in many cases. Thus, we sought an alternative function able to provide an adequate adjustment for most of the segments’ behaviors. In particular, logistic regression emerged as the most convenient solution, offering clear improvements in the categorization of segments for any scenario. In addition, by performing a clustering analysis, we were able to clearly identify three independent categories, whose characteristics are then properly discussed.

The rest of this paper is organized as follows: Section 2 presents some related work regarding studies that predict traffic behavior using different approaches. Section 3 provides information on the SUMO and DFROUTER tools, along with our iterative heuristic. Section 4 describes the methodology that has been used to achieve the desired per-segment traffic modeling, starting from induction loop data, and ending in a classification of the different street segments. In Section 5, we propose an equation of the sigmoidal family to predict the behavior of the street segments that best fit with a logistic regression. In Section 6, we analyze the behavior of traffic congestion through the logistic regression in the city of Valencia and in a specific area of the same city, including clustering results. Finally, Section 7 concludes the paper and discusses the relevance of the results obtained together with their potential regarding future traffic management systems.

## 2. Related Work

In the last decades, many researchers have developed a wide range of predictive models of vehicular traffic from many perspectives. Zhang and Rice [14] proposed a method to predict travel times using a linear regression assuming a linear combination of covariates, and having a simple structure. On the other hand, Guo et al. [15] proposed using an adaptive Kalman filter to predict traffic. They used time series methods to know the future of traffic parameters, and to update selected stable variables. Van Hinsbergen et al. [16] proposed to use a Localized Extended Kalman Filter by solving the slowness problem presented by these filters in large networks; in addition, they use local filters to correct the state of proximity detectors, whose estimations are based on local and noisy sensor data, to predict the state of the traffic.

Another work presented by Min and Wynter [17] develops a methodology to predict traffic based on time-series, where the extension of the methodology has spatial and temporal interactions that are dependent on road traffic context. Costa et al. [18] described the components of a Traffic Telco Big Data architecture for the analysis and prediction of road traffic at a macro level, using the existing infrastructure as a flow of data available in the telecommunications architecture.

Some works dealing with traffic prediction involve learning algorithms such as fuzzy logic [19], although facing some problems such as low accuracy and efficiency. For instance, Onieva et al. [20] presented a case study in which an automated vehicle must cooperate with a driver to achieve cross-road maneuvers without risk, and developed a three layer hierarchical fuzzy rule-based system. The first layer detects the type of maneuver that is needed, the second layer is the appropriate speed to cross an intersection, and the third layer is the actual speed of the vehicle. Hodge et al. [21] presented a binary neural network algorithm for short-term traffic flow prediction using univariate and multivariate data from a single traffic sensor with temporal delays, and combining information from multiple traffic sensors with time series prediction or spatial-temporal lags. Habtie et al. [22] presented an approach to estimate the state of road traffic using the existing cellular network as a source of traffic data, and use a model for estimating the state of the neural network. Porikli and Li [23] trained a set of Hidden Markov Model chains corresponding to five traffic patterns (stop, heavy congestion, open flow, moderate, and empty congestion), and then used a Maximum Likelihood criterion to determine the state of the separated Hidden Markov Models. Differently from previous works, Kunt et al. [24] focused on predicting the severity of motorway traffic accidents by employing twelve accident-related parameters as input to an artificial neural network, a genetic algorithm, and pattern search methods.

Sananmongkhonchai et al. [25] proposed an algorithm based on cells to predict the travel time, estimating the traffic conditions when having multiple GPS receivers integrated in taxis. However, GPS accuracy depends on additional factors such as satellite geometry, signal blocking, atmospheric conditions, and receiver design features. In addition, other studies (e.g., [26,27]) involve vehicle probes for the prediction and detection of accidents in an automated manner. The problem of using raw probe data is that the estimation accuracy is primarily based on driver behavior.

Previous works have developed a wide variety of traffic prediction models from different perspectives, based on either statistical methods or computational intelligence. However, they have drawbacks as these models are developed with synthetic data, assuming traffic conditions, and without a realistic traffic flow.

This paper proposes an equation belonging to the sigmoid function to properly characterize the travel time behavior of different streets based on the measured number of vehicles ahead, using as input real traffic data. Additionally, we classify vehicular traffic behavior for the different streets segments of the city of Valencia through a clustering algorithm, and use a statistical technique to properly describe the clustering dataset.

## 3. Overview of the Simulation Tools Used

In this section, we provide some details about the SUMO traffic simulator [12]. We also introduce DFROUTER [9], and briefly explain how it allows us to generate a traffic matrix detailing origins and destinations (typically known as O-D matrix) for SUMO based on induction loop data.

### 3.1. SUMO

Usually, the traffic model consists of obtaining some variables, such as the departure and arrival times, the route followed by the different vehicles, and the streets that vehicles pass through.

SUMO [12] performs the simulation of vehicular mobility through a detailed microscopic modeling of cities and vehicles. In fact, being an open source simulator, it is constantly being improved, and is widely accepted by the scientific community. Its features include support for different map formats including OpenStreetMap, importing road networks in multiple formats, and generating routes with multiple sources. In addition, it offers high-performance simulation capabilities through the TraCI interface, such as interactive access to the simulation of route traffic, retrieving values from simulated objects and manipulating their behavior “online”, and enabling many more features when coupled with another simulator such as OMNeT++ [28].

The flow of traffic is simulated microscopically, meaning that each vehicle movement within the road network is individually modeled. This feature allows us to constantly know each vehicle’s location, speed, acceleration, time of departure, and time of arrival. By default, each time step has a duration of one second, which allows a discrete simulation of continuous mobility in space.

### 3.2. O-D Matrix Generation with DFROUTER

One of the packages included by the SUMO simulator version 0.20.0 is the DFROUTER tool. This tool has been designed for road scenarios based on the main idea that roads are equipped with induction loops that allow measuring the inflow and outflow of the roads. DFROUTER can reconstruct the number of vehicles and routes to be injected into the simulator of the road network, based on the data obtained from induction loops such as the number of vehicles, flows, and speeds, to achieve the desired O-D traffic matrix. In other words, this tool, starting from induction loop counts for the different streets of a city, is able to estimate the possible vehicle routes that match such input.

**Figure 1:**
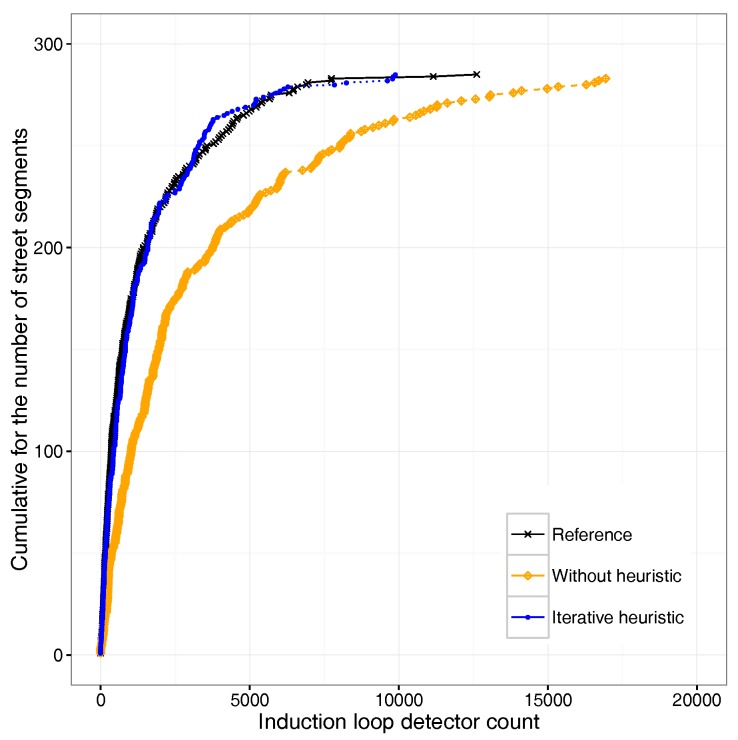
Traffic flow modeling for Valencia. Results with and without our iterative heuristic [10].

In a previous work [7], we  used induction loop data provided by the City Hall of Valencia, Spain, as input to DFROUTER. These data sets were generated by 520 induction loop detectors deployed throughout the city, and correspond to the rush hour (between 8:00 and 9:00 a.m.) for a typical Monday. In that work, we noticed that there was a significant mismatch between the traffic generated and the original data. We then introduced an iterative heuristic that compensates for this error by refining the output provided by this tool in order to achieve an O-D matrix that resembles the real traffic distribution. Figure 1 shows that, as a result, the output of the DFROUTER using our iterative heuristic process achieves a high level of matching with the reference data, resulting in an error lower than 0.0001%, and thus being significantly better than the initial DFROUTER output.

## 4. Methodology

**Figure 2:**
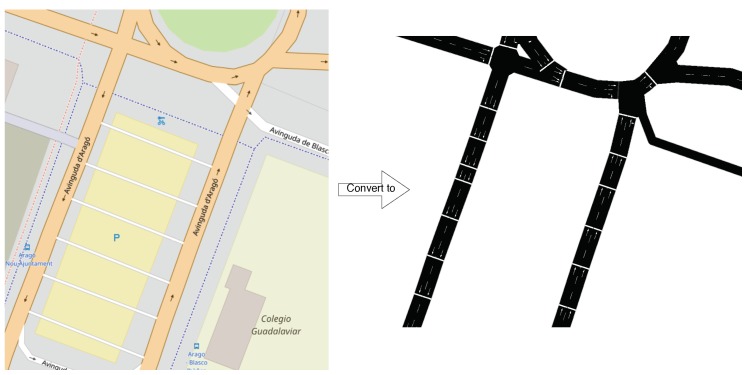
Example of unnecessary street partitioning.

In this section, we describe the procedure followed to characterize the traffic in the city of Valencia, Spain, from a microscopic perspective, and starting from OpenStreetMap road layouts. In particular, our goal is to characterize individual street segments in terms of average travel times experienced by vehicles for different degrees of congestion, being the latter estimated based on the number of vehicles found ahead by a vehicle when just entering a segment. To achieve this goal, we found necessary to first perform some preprocessing, as in many cases the presence of micro-segments (streets unnecessarily partitioned in many short segments) impeded an adequate analysis of the behavior of vehicles traversing particular streets, as we can see in Figure 2. Then, we used the SUMO tool coupled with the OMNeT++ simulator to study the traffic flow for the entire city of Valencia based on a realistic traffic trace, as described in the previous section.

Below, we describe the methodology followed to characterize and predict traffic for the different street segments. Our proposed methodology is the following: first, we unify segments whenever required; next, we predict the number of vehicles in each segment; and, finally, we characterize the different street segments through a regression analysis, which is later complemented by a clustering of these results to achieve a proper classification. Below, we detail the algorithms we proposed to unify segments, to predict the traffic time, and to characterize the different segments according to vehicle travel times.

### 4.1. Unifying Segments

Usually, when the city map is converted to a format accepted by SUMO for simulation, certain characteristics of the map must be eliminated, such as bicycle paths, pedestrian paths, train tracks, etc. This conversion has a drawback because it causes the streets to be intercepted by other ways, different from those used by vehicles, and the SUMO simulator acts by partitioning those streets. In many cases, the unnecessary partitioning of streets causes inconveniences such as:

1. Streets are partitioned into tiny segment sizes, often measuring less than 7.5 m (size of a vehicle plus inter-vehicular security gap).
2. Such small sizes do not allow to characterize the segment profile correctly.
3. Inconsistent graphs are obtained when applying the regression analysis to predict traffic behavior.

To understand the solution adopted to address this issue, we should mention that the IDentification (ID) that represents a street segment is composed of two parts, where the first part is the code identifying the street, and the second part is the sequential code assigned by SUMO to each street partition. The proposed procedure tries to unify those street segments whenever possible if certain conditions are met. To achieve this, it is necessary to have a dictionary that stores all the segments without intersections, as well as a dictionary that stores all the connections of those segments. Then, we must compare each connection of the different segments to determine whether they meet the conditions required to perform segment unification. In particular, the conditions that a set of segments must meet to be reunified are the following:

1. The street to be reunified must be a set of partitioned segments.
2. The adjacent segment should not have another segment that intersects it.
3. The street ID codes must be the same for segments to be reunified.
4. Segments to be reunified must have consecutive numbers in their sequential part of the ID.

Once all these conditions have been met, at least two segments can be renamed and unified to achieve the correct prediction of the traffic, according to Algorithm 1.

**Algorithm 1** Segment reunification strategy.**Require:** Road Network file, edges files**Ensure:** Reunified segment file1:edgeConnectedNoIntersection[]← dictionary that stores all edges without intersections2:**for all** edge **in** Road Network file **do**3:    edge_id← store the edge id of the road network file4:    connections[]← dictionary that stores all connections for that edge id5:    **for all** connection **in** Road Network file **do**6:        connection_from← store the edge id(from) of the road network file7:        connection_to← store the edge id(to) of the road network file8:        **if** (connection_from=edge_id) **and** (connection_to notin connections) **then**9:           connections[edge_id]←connection_to10:       **end if**11:   **end for**12:   **if** edge_id partition = TRUE **then**13:       lenEdgeConnect← length of dictionary in a specific edge id14:       street_id← code of the street15:       **for** i=0 **to** length(connections[edge_id]) **do**16:          **if** (lenEdgeConnect=1) **and** (connections[edge_id][i] partition = TRUE) **and** (street_id in edge_id) **and** (street_id in connections[edge_id][i]) **then**17:              edgeConnectedNoIntersection[edge_id]←connections[edge_id][i]18:          **else**19:              edge_id← has some intersecting segments, cannot reunify20:          **end if**21:       **end for**22:   **else**23:       edge_id← is not split, reunification is unnecessary24:   **end if**25:**end for**

### 4.2. Per-Segment Travel Time Prediction

In this section, our goal is to predict the travel time associated to each segment for different degrees of congestion, being the latter measured as the number of vehicles located in the segment just before a new vehicle enters it ($\nu_{n}$). We propose Algorithm 2 to achieve this prediction; in particular, we have to consider the input time ($t{}_{in}^{\nu }$) and the output time ($t{}_{out}^{\nu }$) of the vehicle in the segment, as well as the number of lanes of the segment ($l_{n}$) where the vehicle is traveling. Input times ($t_{in}$) for vehicles entering a segment on lane $l_{n}$ are registered in matrix ($l_{n}$,$t_{in}$), while output times ($t_{out}$) for vehicles leaving the segment at lane $l_{n}$ are then registered in matrix ($l_{n}$,$t_{out}$). To avoid erroneous values for the travel time prediction associated to each segment, it is necessary to perform a sorting of the matrix by $t_{in}$. The number of vehicles in the segment just before a vehicle joins it ($\nu_{n}$) will increase as long as $t{}_{in}^{\nu }$ is less than $t{}_{out}^{\nu }$, and both refer to the same lane. Then, the travel time of each vehicle in the segment will be obtained (Δt). As a final step, according to Algorithm 2, an average of the travel times ($\bar{\Delta t}$) associated to different degrees of congestion (number of vehicles in the segment before a new vehicle enters that segment) will be included in a file, along with the number of vehicles in that segment (ν).

**Algorithm 2** Extraction of travel times vs. load samples.**Require:** Reunified segment file, Segment-info files**Ensure:** Statistical learning by segment files1:**for** segment **in** Reunified segment file **do**2:    segmentConnected[]← vector that stores all edge ids connected3:    **for** *s*=0 **to** length(segmentConnected) **do**4:        segment_info← Read lines segmentConnected[s] in Segment-info files5:        segmentSorted[][]← sort_by_$t_{in}$(segment_info)6:        **for** $t_{in}$=0 **to** length(segmentSorted) **do**7:           $\nu_{n}\leftarrow$ number vehicles per segment in each lane8:           **for** $t_{out}=t_{in}$ **to** $t_{out}>=0$ **step** − 1 **do**9:               **if** $\left(segmentSorted\left[t_{in}\right]\left[l_{n}\right]=segmentSorted\left[t_{out}\right]\left[l_{n}\right]\right)$ **and** $\left(segmentSorted\left[t_{in}\right]\left[t{}_{in}^{\nu }\right]<=segmentSorted\left[t_{out}\right]\left[t{}_{out}^{\nu }\right]\right)$ **then**10:                   $\nu_{n}=\nu_{n}+1$ increase the number of vehicles if the condition is met11:               **end if**12:           **end for**13:        **end for**14:        $\bar{\Delta t}\leftarrow$ average of the travel times for different degrees of congestion15:        ν← number of vehicles in the segment before a new vehicle enters the segment16:    **end for**17:
**end for**



### 4.3. Segment Behavior Characterization with Polynomial Regression

Once the process described above to estimate travel times in a segment for different degrees of congestion is completed, the next step is to characterize and classify the behavior of the different segments. In particular, we seek to determine the relationship between the number of vehicles in a segment (*x* values), and the average travel time of vehicles (*y* values) for each particular segment. To achieve this goal we perform regression to obtain the best curve fit describing the nonlinear relationship between segment congestion and travel time. Because traffic theory, in general, considers that the relationship between traffic load and travel time tends to vary quadratically [13], to perform the fitting, we used function $f\left(x\right)=ax^{2}+t_{ff}$ in a first attempt. Thus, the chosen expression is able to adequately represent this parabolic behavior starting from the free flow travel time (no congestion), represented by constant $t_{ff}$ in this function, and then increasing as the number of vehicles ahead in a segment (represented by *x*) increases.

**Figure 3:**
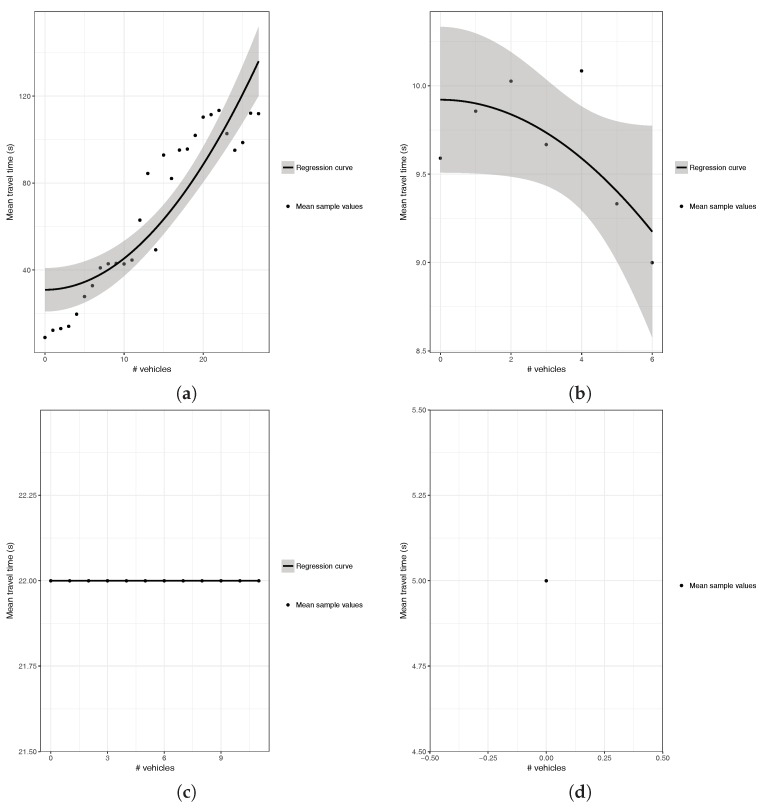
Segment Classification: (**a**) increasing; (**b**) decreasing; (**c**) constant; and (**d**) unique.

Once the regression results for all the segments tested where obtained, we observed that the expected quadratic behavior was indeed taking place in many of the segments, although other special cases were also detected. Figure 3 presents different representative cases corresponding to the patterns we observed. The first class of polynomial regression curves, which illustrates the expected pattern according to traffic engineering theory, is shown in Figure 3a. As can be seen, when a vehicle entering a segment finds many vehicles ahead, it will, on average, experience much higher travel times, with differences up to 1000% being possible and expected. However, other patterns were also obtained, as traffic flow properties also cause other types of behavior to take place, especially when modeling a very large city such as Valencia. For instance, the second class of curve represents a behavior that is just the opposite compared to the previous one. As shown in Figure 3b, this kind of curve shows an increase followed by a decrease in the time traveled as we increase the number of vehicles ahead. Such behavior is explained by different factors, including the departure of vehicles from the segment, as they turn to join other segments, and, more important, the presence of traffic lights that tend to accumulate vehicles on the segment, being that vehicles finding many vehicles ahead usually means that the accumulation period was long, and the semaphore is about to turn green.

In addition to the two types of behavior described above, there are also other cases taking place, as exemplified in Figure 3c,d. Regarding the behavior observed in Figure 3c, we can see that the travel time remains constant regardless of the number of vehicles in the segment, typically meaning that there are no semaphores (areas in the periphery of the city), no junctions, and that the capacity of the segment is much higher than the number of vehicles detected during the simulation (typically multi-lane segments), and so congestion effects are not perceived. Finally, the behavior of the last group, as described in Figure 3d, corresponds to one-lane segments rarely visited by vehicles according to the traffic patterns used as input. Thus, we do not have enough information to characterize the behavior of the segment at higher loads, as the traffic flow levels are minimal.

Overall, we find that the characterization of the behavior of the segments in this scenario has not been done accurately in many cases where the vehicular load remained low. Thus, we deem it appropriate to saturate with additional vehicles the different segments of the city, and then improve our awareness regarding the behavior of all segments with the different degrees of congestion. In addition, we also know what happens with the second class of the curve shown in Figure 3b, which represents an abnormal situation according to the traffic theory. To achieve this goal, it is necessary to use the Equation $\tau_{s,n}=\frac{\sigma_{s}}{\omega_{s}}\cdot \varphi_{n}+\lambda$ presented in a previous work [11], to gradually increase the number of vehicles in our reference traffic scenario for Valencia until a higher saturation level is reached. This is achieved by assigning a variable number of additional vehicles to be injected at each traffic source location. The variation of the total number of vehicles injected in this scenario ranges from 2271 to 34,065.

**Figure 4:**
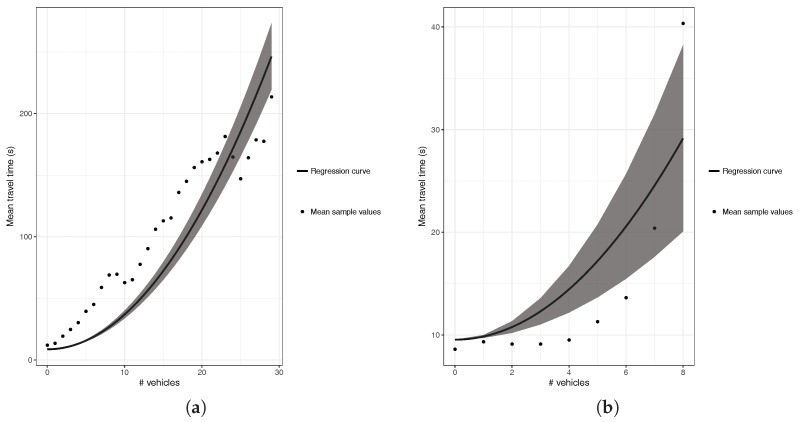

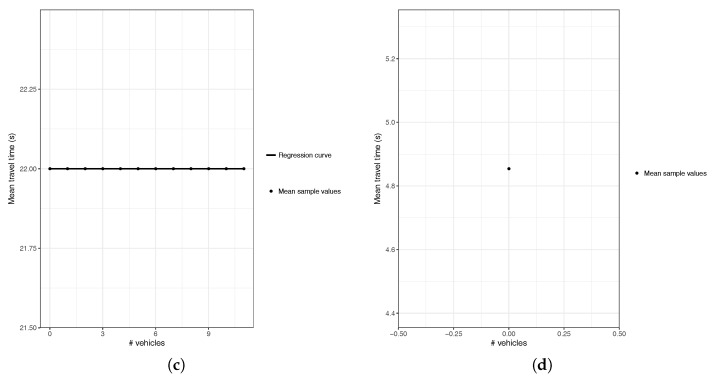
Segment Classification with uniform traffic load regulation: (**a**) increasing; (**b**) behavior of a segment previously showing a decreasing behavior; (**c**) constant; and (**d**) unique.

The effect of saturating our scenarios with more vehicles is significant, as the prediction of the resulting traffic through the quadratic regression now suppresses the second class (decreasing) detected above, being that only three types of behavior remain, as shown in Figure 4. The first type, called incremental, agrees with general traffic theory, merely stating that the greater the number of vehicles ahead, the greater becomes the travel time along a street. As shown in Figure 4b, segments that previously had a decreasing trend now clearly have an increasing behavior, therefore meeting traffic theory. However, the segments belonging to the constant classification continue to persist for the same characteristics (see Figure 4c), as the location of the segment in areas of the periphery of the city, which do not have links with other streets, remain mostly uncongested. In addition, the segments having a unique behavior also persist, as they are in general very small segments only sporadically visited by vehicles (see Figure 4d).

A particularity that we have observed in the increasing behavior (see Figure 4a,b) when applying the quadratic regression to the segments of the city is the fact that they often fail to adjust properly to the relation of the number of vehicles ahead and the time traveled. This causes a high standard error, meaning that the regression does not adequately represent the actual behavior of the segment. Due to this inconvenience, we considered necessary to find another type of regression that better adjusts to the behavior of the segments of the city to reduce the prediction error.

## 5. Proposed Predictor of Vehicular Travel Times

In Section 4.3, when analyzing the behavior of the traffic in a city following general traffic theory criteria, we can observe that the polynomial regression fitting results can be deemed inadequate for most segments. For this reason, our aim is to find an alternative mathematical function that better adapts to the behavior that characterizes the different segments of the city. In this paper, we propose using a mathematical function that belongs to the logistic family of functions. In particular, we picked the sigmoid function to represent the growth patterns detected in our data set. Thus, we have a logistic regression to predict the outcome of a variable that can adopt a limited number of categories based on independent or predictor variables, and this kind of regression is used to model the probability of an event that occurs as a function of other factors [29]. To this end, we rely on the simple sigmoid function defined by the following mathematical expression:

$$f\left(x\right)=\frac{1}{1+e^{-x}}$$

Since we have to adapt the curve of this function to the travel time in free-flow conditions (zero vehicles ahead), we add a parameter *b* to the equation, together with a second term of the initial function to make this possible. In particular, parameter $t_{ff}$ allows defining such free-flow travel time:

(1) $$f\left(x\right)=\frac{1}{1+e^{b-x}}-\frac{1}{1+e^{b}}+t_{ff}$$

Finally, to be able to adapt Equation (1) to meet the actual maximum value for the travel times measured, we extend this equation by adding parameter *a*, and determine its corresponding displacement in the axis of the abscissa with the parameter *c*, as shown in Equation (2).

(2) $$f\left(x\right)=\frac{a}{1+e^{b-\frac{x}{c}}}-\frac{a}{1+e^{b}}+t_{ff}$$

This equation is the one adopted for the regression analysis that follows.

## 6. Traffic Congestion Behavior Analysis

In the previous section, we have presented a function that better adapts to the behavior of the segments of our target city through a logistic regression. We now study the behavior of the city under different degrees of vehicular congestion. This is achieved by regulating the number of vehicles in our simulation following the same method described earlier, based on taking the standard number of vehicles during rush hours and injecting an extra number of vehicles in each street segment, according to Zambrano-Martinez et al. [10], and using our Equation $\tau_{s,n}=\frac{\sigma_{s}}{\omega_{s}}\cdot \varphi_{n}+\lambda$. In particular, we vary the total number of additional vehicles injected into the simulation from 2271 to 34,065. Notice that the total number of segments for this scenario is 9859.

Once the simulation results were obtained, the processes described in Algorithms 1 and 2 were performed. The process of characterization and classification of the behavior of the different segments in the city was determined by the relationship between the number of vehicles existing in the segment (abscissa axis) and the average travel time in that segment (ordered axis). When concluding with the logistic regression based on Equation (2), for all the segments, we can observe similar patterns as in the quadratic approach, with most segments showing an increasing behavior, and few segments showing a constant/unique free-flow characteristic.

### 6.1. Validation of the Logistic Regression

**Figure 5:**
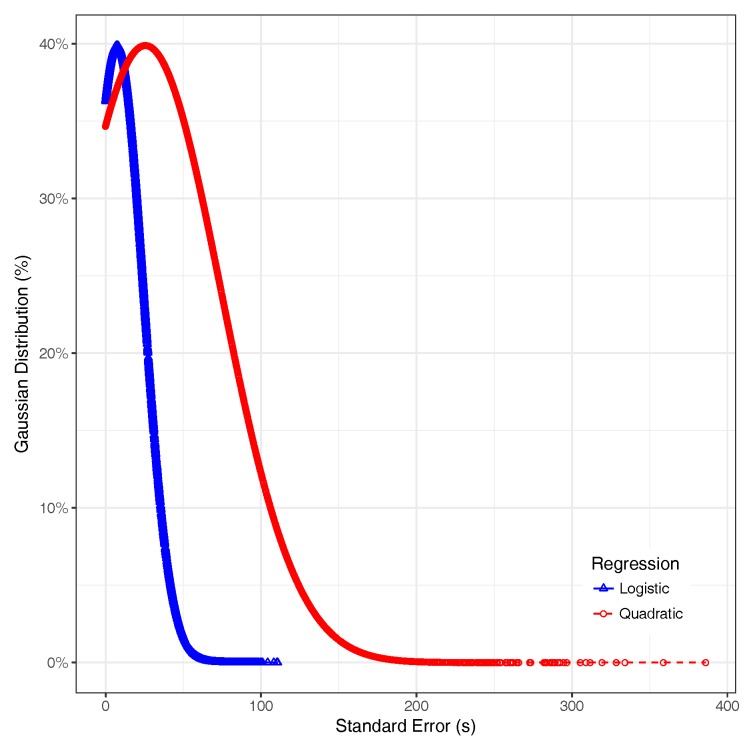
Standard error of regressions.

To achieve the characterization and classification of the segments, we performed the logistic regression using Equation (2), which gives a better fit than using the second order polynomial function, substantially reducing the mean standard error from 25.6477 to 7.3587, as shown in Figure 5. In detail, we found that most of the standard error values for the logistic regression remain below 25 s, and the segments with a standard error greater than 50 s represent less than 1% of the segments of the vehicular network of the city. On the other hand, the standard error associated to the quadratic regression can go beyond 300 s, and the error associated to most street segments is greater than 50 s.

**Figure 6:**
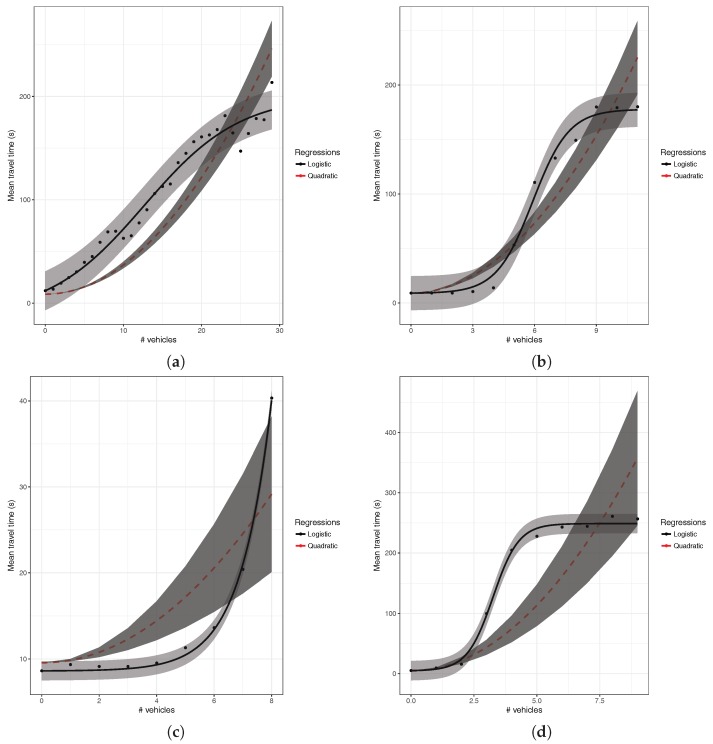
Several examples where the logistic regression outperforms the quadratic regression.

Figure 6 shows some examples that illustrate how the logistic regression was able to significantly improve the curve fitting error when compared to the standard approach, based on quadratic regression. Therefore, it becomes clear that, by using a logistic regression to characterize the behavior of the segments, the travel time predictions for different travel loads are significantly improved.

### 6.2. Clustering Results with Logistic Regression

In the previous section, we highlighted the benefits of adopting a function belonging to the logistic family r to properly represent the behavior of the different streets in a city in terms of their travel time characteristic for different traffic loads. Once this step was completed, we then proceeded to perform an appropriate classification of the different streets according to their pattern. Thus, we applied a clustering technique to correctly classify the different groups of streets according to their behavior.

The number of street segments for this scenario is 9859, as referred to in Section 6. The representation of each characterized group was done using a learning machine technique called K-means [30], and we used the parameters of the logistic regression in Equation (2) to perform an automatic categorization of the different street segments, assigning each segment to a specific category according to its behavior. With respect to the parameters used as input for the characterization of the segments, we have parameter *a*, which allows us to discriminate between increasing and constant trends, as it represents the amplitude of the regression curve in the ordinate axis. Likewise, parameter *c* gives us the characteristic of the maximum extension of the sigmoid curve on the abscissa axis, which represents the number of vehicles in the segment. Another input parameter for the classification procedure is $t_{ff}$, which represents the travel time on a segment when there are no vehicles ahead, and that helps us to distinguish segments according to their free-flow speeds. Finally, we use a parameter called $f{\left(x\right)}_{max}$ that represents the highest travel time associated with a particular segment.

**Figure 7:**
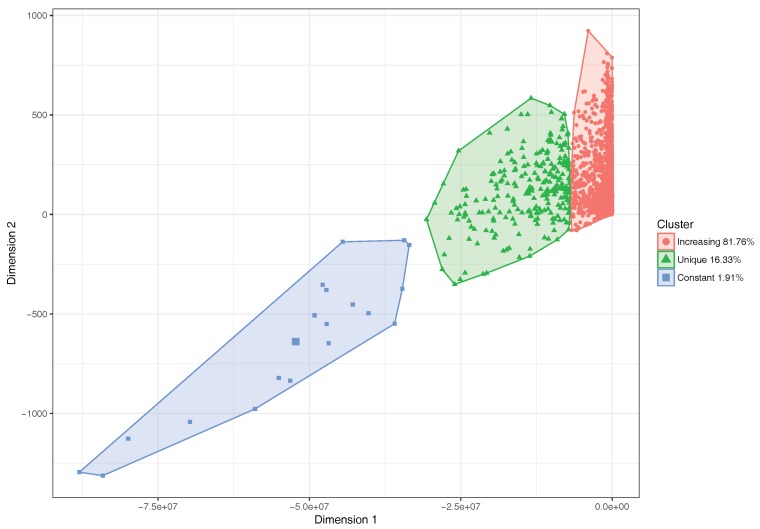
Segment classification with logistic regression by clustering.

The K-means clustering method clearly identifies three clusters. We then used the Principal Component Analysis (PCA) [31] procedure to reduce the graphical representation of the four input parameters to two dimensions, and thus be able to understand the classification of the segments in a visual manner. Figure 7 shows the result of applying K-means clustering to the city of Valencia. We observe that the percentage of segments with the expected increasing behavior is 81.76%. Likewise, we can observe that the percentage of segments within the constant category is 1.91%, while 16.33% of segments of the city belong to the unique segments family, which are in general quite small segments than remain despite applying our segment reunification algorithm.

**Figure 8:**
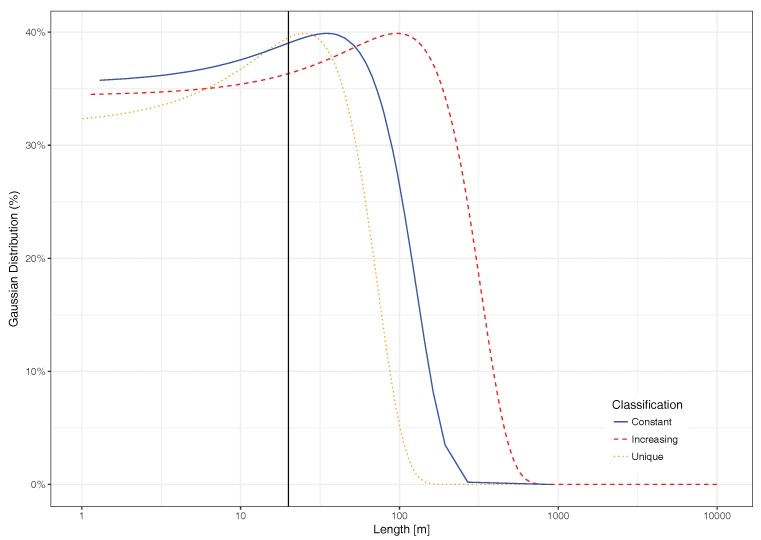
Normal distribution of segments length.

In general, the presence of segments characterized by a constant value, even though many vehicles are injected into the segments of the city, can be a problem in the sense that such behavior is not realistic. On the other hand, the unique segments do not reflect the effects of traffic saturation because many of them still represent very small partitions, as in the case of a roundabout that fails to accomplish the conditions of Algorithm 1, impeding several very small segments from being unified. For this reason, a more in-depth analysis of the actual segment lengths has been performed. We found that there were segments having a length that is less than the length of a standard vehicle. Thus, for our study and overall purpose of predicting traffic delays, such segments are useless. In fact, each segment whose size is inferior to, at least, the length of the vehicle plus the inter-vehicle space, can be discarded. To achieve this, we opted for the criterion proposed by Cal y Mayor and Cárdenas [32], which is a theory of vehicular flow that accounts for vehicle flow, speed, density, interval, and the spacing between vehicles. According to these authors, the fundamental equation of the vehicular is able to relate a constant approximate speed, the average free time interval between two vehicles, and their average spacing. Thus, we applied it to our scenario, and the obtained result is very close to 20 m in length for any segment, which gives us a threshold to filter out any segments that measure less than 20 m. Figure 8 shows that segment lengths of the different categories follow a Gaussian distribution, and we can visually perceive the effect of such filtering threshold. In fact, a high percentage of segments belonging to the unique family are below this threshold. With respect to the other two categories, only a small percentage of these segments fall below the threshold. Thus, we consider it to be adequate for our purposes.

**Figure 9:**
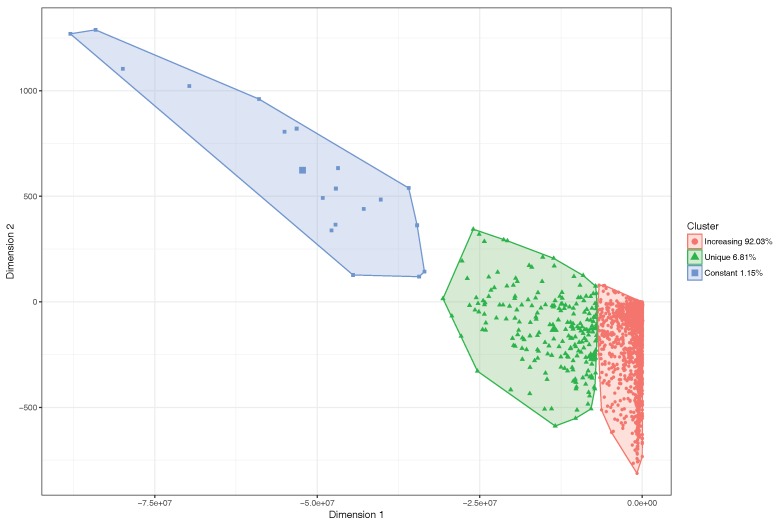
Segment classification through clustering for the logistic regression, after applying the filtering threshold.

After filtering out these small street segments, we proceed to identify what is now the actual percentage of segments that belong to each category by again applying the clustering algorithm, and retrieving its corresponding visual representation through the PCA procedure. Figure 9 shows that the percentage of segments in the first category now grows up to 92.03%, and that the percentage of segments in the unique category drops to only 6.81%, which is a significant decrease. Finally, a similar number of street segments (1.15%) remains in the constant behavior category.

**Figure 10:**
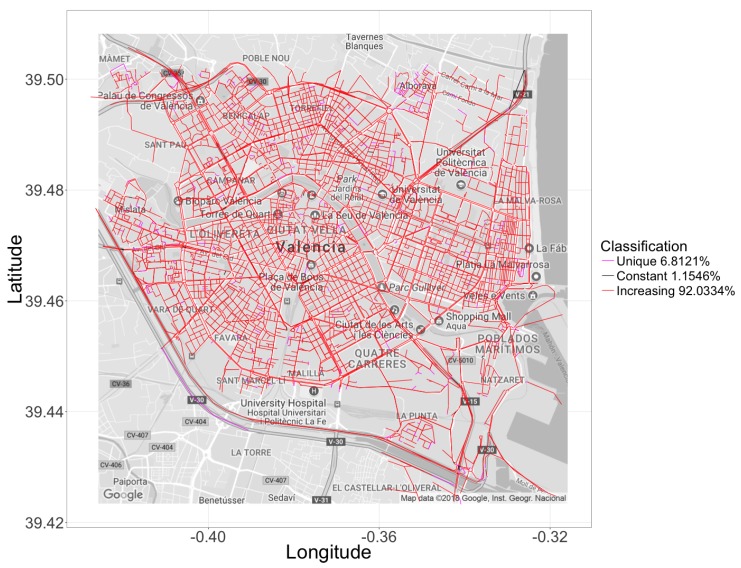
Geographical distribution of the segments classification.

Figure 10 provides an overview of the actual geographical distribution of the streets segments belonging to each of these categories in the city of Valencia. As expected, all major city arteries belong to the “increasing” category, being that only very small and remote segments, located in secondary streets, belong to the two other categories.

### 6.3. Hotspot-Based Traffic Congestion Behavior

In this section, we focus on situations where a public event reaches a high number of people for a restricted area, causing the city to experience a heterogeneous congestion effect. For this scenario, which we named “hotspot”, we have chosen an area of 270 m radius centered on the Mestalla football stadium, and that has 106 different predicted routes passing nearby. The strategy to achieve our goal is to gradually inject vehicles in this scenario from 100 to 10,000, which are scattered throughout an area including a total of 887 segments. Thus, this scenario combines both “regular vehicles”, just passing by that area as in a regular day (departing and arriving), and “hotspot vehicles”, which depart from the hotspot area and move to any random destination. Our goal is to study the effects of such asymmetric congestion states when compared to the situation studied earlier, where congestion is more homogeneous. In particular, we want to show if such localized traffic congestion can generate conditions that allow performing a better characterization of the different street segments in terms of their associated travel times prediction curve.

**Figure 11:**
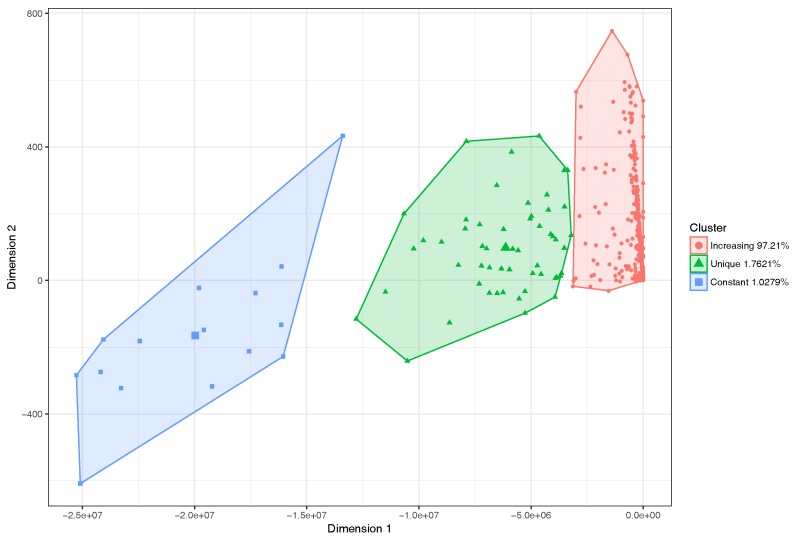
Segments classification through clustering for the logistic regression in the hotspot scenario.

**Figure 12:**
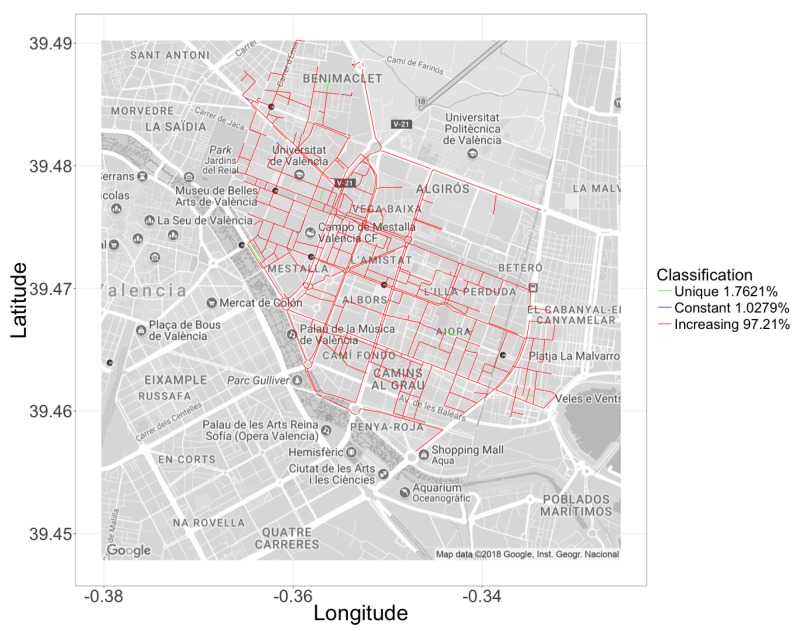
Geographical distribution of the segments in the different clusters for the hotspot scenario.

For this scenario, we applied Algorithms 1 and 2 to the segments that belong to the hotspot, and obtained the travel times prediction. Afterwards, we applied the logistic regression (see Equation (2)), and obtained the behavior of the different street segments. Again, we found that the behaviors of the segments within this area belong to the same three categories reported before: increasing trend, unique value, and constant value. As explained previously, the presence of micro-segments in these scenarios persist as they cannot be reunified since they fail to meet the four conditions presented in Section 4.1. Thus, we again applied a filter to the length of these segments to discard excessively tiny ones that are irrelevant in the scope of our case study. We then proceeded to perform the automatic classification through the clustering algorithm together with the PCA, to visualize those groups in a two-dimensional space. As shown in Figure 11, the clustering technique now shows that 97.21% of the street segments belong to the main category, meaning that the majority of street segments can now be properly characterized in terms of travel time behavior for different vehicle congestion levels. The two remaining groups are now associated to a very low percentage of segments: 1.7621% for a the unique value category, and 1.0279% for the constant time case. Likewise, we can observe in Figure 12 the geographical location of the segments within the studied scenario, differentiated according to their behavior. We can see that the segments for which we have a poor delay characterization (unique/constant value cases) are indeed not quite relevant from a global perspective, being that for the majority a clear view of the travel behavior can be obtained, and such per-segment characterization used as input to a larger route planning system.

## 7. Conclusions and Future Work

Having a realistic traffic model for a specific target city is a key requirement to obtain meaningful simulation results when issues such as traffic density and traffic patterns can have an impact on the conclusions derived from experiments. Achieving such realistic models typically requires describing traffic in terms of Origin–Destination (O-D) matrices. In addition, if aimed at developing advanced traffic management solutions, it becomes further necessary to have a more in-depth understanding of how traffic is distributed in a particular city, which basically requires performing a correct analysis and classification of such traffic.

The starting point of this paper was a realistic traffic model for Valencia derived in a previous work. Then, the contribution of this paper was the characterization of all the street segments in Valencia in terms of travel times when vehicles face different degrees of congestion. To achieve this characterization, we started by processing the map of the target area in order to merge segments of the same street whenever unnecessary fragmentation was detected and could be reversed. Then, we performed simulation experiments using SUMO to retrieve the travel times of vehicles when facing different degrees of traffic saturation on the traveled segment. Finally, using different regression strategies, we performed curve adjustment to obtain an expression that allowed us to characterize these travel times.

Once all segments where characterized, our next contribution was to apply clustering to automatically classify segments according to their travel time behavior. In particular, we applied the K-means technique to generate the clusters, followed by a Principal Component Analysis to extract the main clustering features that enable visual representation. The results of the clustering process clearly define three categories: segments with incremental traffic delays (the majority), segments with constant delays (typical loads do not cause congestion), and single value results, corresponding to small segments rarely visited by vehicles. We complemented this study with an analysis of segments lengths to filter out segments that are too small, and so not representative for our traffic analysis. We also showed how the street segment characterization could be improved by causing very high congestion levels in a hotspot scenario, situation where more than 97% of the segments could be characterized adequately.

As future work, we plan to develop a centralized traffic management platform that, based on the per-segment travel delay characterization provided in this paper, is able to globally minimize vehicle travel times by accounting for congestion, and to perform load balancing. Once the platform is developed, the impact of having different types of vehicles will be studied, with their respective characteristics such as maximum speed, allowed traffic schedules, and specialized lanes for public service vehicles. This will allow us to know the efficiency of the platform when accounting for the various types of vehicles available (e.g., buses and trucks) on the overall traffic flow.
